# Remediation of adult black dental stains by phototherapy

**DOI:** 10.1038/s41405-018-0001-9

**Published:** 2018-04-11

**Authors:** M. A. Albelda-Bernardo, M. del Mar Jovani-Sancho, V. Veses, C. C. Sheth

**Affiliations:** 1 Department of Dentistry, Faculty of Health Sciences, Universidad CEU Cardenal Herrera, CEU Universities, Moncada, 46113 Valencia Spain; 2 Department of Biomedical Sciences, Faculty of Health Sciences, Universidad CEU Cardenal Herrera, CEU Universities, Moncada, 46113 Valencia Spain; 3 Department of Medicine, Faculty of Health Sciences, Universidad CEU Cardenal Herrera, CEU Universities, Moncada, 46113 Valencia Spain

## Abstract

**Introduction and aims:**

This study investigates the effects of the application of antimicrobial phototherapy on black-stained tooth surfaces. Key variables were area, color and time taken to reappearance of black stain following dental prophylaxis with and without antimicrobial phototherapy. Differences in bacterial composition of black stain (specifically *Aggregatibacter actinomycetemcomitans*, *Tannerella forsythia*, and *Porphyromonas gingivalis*) were analyzed.

**Material and methods:**

The phototherapy device used was GLO™ Science LLC, New York, America, emitting light at 475 nm and a power of 3 W. Light was applied in 2 cycles of 8 min on 31 volunteers. Microbial identification was carried out on DNA extracted from black plaque. This study is registered with ClinicalTrials.gov, number NCT03309748.

**Results:**

Following antimicrobial phototherapy, 64.5% of patients displayed reduced pigmentation area. Plaque color was lighter in 48.4% of subjects. Pigmentation area and depth of color returned to normal levels during the course of the study. Colonization by the three bacterial species decreased, although the changes were not statistically significant. We report a key novel finding showing elevated levels of colonization by *Tannerella forsythia* (83.9%) in adult black stain.

**Conclusions:**

Application of phototherapy results in a reduction in area, color and bacterial colonization of black plaque in adults. The changes were not found to be statistically significant, perhaps owing to the low illumination power of the home-whitening device. For the first time, we document the elevated presence of *Tannerella forsythia* in adult black stain. We also demonstrate the potential application of a commercially available home-whitening device for black plaque treatment.

## Introduction

One of the most commonly encountered difficulties for the dentist in daily practice is the treatment of chromatic alterations of teeth, both of intrinsic and extrinsic origin. The former, caused by the incorporation of chromogenic materials in enamel and dentin, prior to and following eruption, may result in the application of complex and aggressive treatments for their elimination. In contrast, the latter, caused by the deposition of pigment or detritus on the tooth surface, are cleared relatively easily resulting in less damage to the enamel. Black stain (BS) manifests as dots or black lines distributed in parallel to the gingival margin of teeth commonly in the cervical third, and is firmly attached to the enamel, thereby complicating its removal.^1^ BS has a reported prevalence between 1% and 20%, and it can occur at any age, although its prevalence appears to peak in childhood.^2^ BS is considered to be a special form of dental plaque with a tendency for calcification.^3^

The etiology of the process of BS formation is unclear. Early literary evidence suggested a role for chromogenic bacteria in the production of black pigment. Since then, several studies have tried to identify the microorganisms present in black plaque by classic microbiological and molecular methods, and have demonstrated the presence of *Porphyromonas gingivalis*, *Actinomyces* spp., *Aggregatibacter actinomycetemcomitans*, *Streptococci*, *Lactobacilli*, and *Prevotella* spp.^4–6^ A recent study revealed increased presence of *Actinomyces*, *Cardiobacterium*, *Haemophilus*, *Corynebacterium*, *Tannerella* and *Treponema* in plaque samples of children with BS.^7^ To the best of our knowledge, there have been no corresponding studies in adult patients.

The standard treatment practice for BS is dental prophylaxis, however this is often harmful to the enamel surface and can result in tooth sensitivity.^8^ In this study we aim to explore the application of antimicrobial phototherapy (aPT) for the treatment of adult BS. aPT has demonstrated positive results in the treatment of periodontitis in vivo.^9^ The mechanism of action of this method is defined as the light-induced inactivation of microorganisms.^10,11^ aPT represents a non-invasive, minimally aggressive treatment option. As several of the bacteria that have been identified in black plaque samples are known periodontopathogenic agents, we decided to evaluate the effect of aPT application on the buccal surface of teeth with black extrinsic pigmentation of bacterial origin. The key outcome variables recorded were variations in area and color, as well as levels of colonization by *A. actinomycetemcomitans, P. gingivalis,* and *T. forsythia*. These three microorganisms have been shown to have the strong association with periodontal disease in adults^12^ hence we hypothesize they could also play a role in black stain.

## Methods

### Subjects and plaque samples

In this study BS dental plaque samples from 38 orally healthy Caucasian adult volunteers (24 women and 14 men) were collected and analyzed. Children, pregnant women and individuals completing antibiotic therapy during 15 days prior to the start of the study, were excluded. The study protocol was approved by the Ethics Committee of the University CEU- Cardenal Herrera (Authorization number CEI16_019) and it follows the guidelines in the Declaration of Helsinki. This study is registered with ClinicalTrials.gov, number NCT03309748. The volunteers of the study followed the protocol and gave their written, informed consent.

### Phototherapy device

The phototherapy device used in this study was the GLO Brilliant home use whitening device (GLO™ Science LLC, New York, America), comprising 15 blue light-emitting diodes (LEDs) with a wavelength of 475 nm and a global power output of 3 W, according to manufacturer’s specifications. The light source was capable of irradiating premolar and molar teeth, as well as free gingiva from the buccal side.

### Phototherapy

The device was wrapped in clear film in order to maintain hygiene. The film was discarded between applications and patients. Teeth were irradiated during a single session for 8 min followed by a second cycle of 8 min (total exposure of 16 min for each subject). This protocol was devised based on a previous a PT study in which the investigators applied light for 2 min, twice daily for 4 days for a total of 16 min.^9^ We use the same overall duration of exposure in one session for ease of application for the dental practitioner and improved patient adherence.

### Study protocol

All practical aspects of this study were carried out by a single registered dentist with 12 years of experience working daily in a dental clinic. Therefore, it was possible to avoid inter-operator bias associated with the treatments. The intra-operator bias was assessed by measuring the kappa-index. The date of the last dental prophylaxis session was noted during the study patient’s first visit to the clinic. This was followed by an oral examination and confirmation of the clinical diagnosis of BS.

Diagnostic criteria used were as described by Gasparetto^13^ (a modification of Shourie^14^ and Koch criteria^15^). Briefly, patients’ BS was scored as follows; *score 1*, pigmented spots or incomplete lines parallel to the gingival margin, *score 2*: solid lines pigmented, readily observable and limited to the cervical third of the tooth surface, *score 3*: pigmentation extending beyond the cervical third. The patient’s oral cavity was photographed in sextants, along with a complete picture of the jaw (lingual plane) and the maxillary arch (palatal plane). For bacterial analysis, for each patient, a supragingival plaque sample was collected by scraping with a plastic scaler from buccal tooth surfaces taking care to avoid unnecessary removal of enamel hydroxyapatite.^16^ Prior to scraping, the sampling area was isolated with cotton rolls in order to prevent a cross contamination of microorganisms from saliva.^16^ A total of 1 mg of each collected plaque sample was transferred to sterile endodontic paper points (ISO 50) and stored in sterile 0.5 ml DNA-free and RNA-free Eppendorf tubes.^5^ Finally, a dental prophylaxis was performed to remove the BS.

**Fig. 1:**
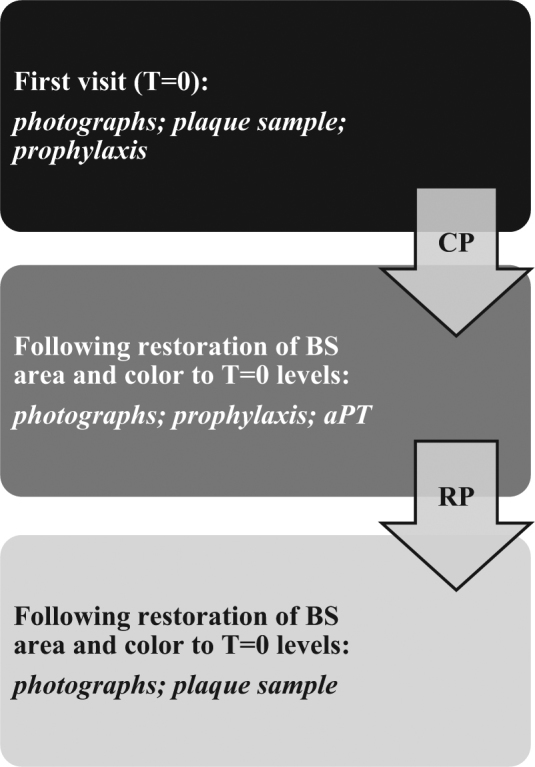
Diagram representing the protocol employed in the study, indicating the comeback period (CP), the restoration period (RP) and the actions taken at each experimental stage. Patients were evaluated monthly during the CP and RP in order to accurately establish the timeline and move patients to subsequent experimental stages; *T* *=* *time*

Patients were reviewed monthly and in each visit, were photographed as previously described, and the level of pigmentation scored according to the mentioned criteria. The monthly visits were continued until the BS score returned to the initial value. This period between the first and the last appointment, was named Comeback Period (CP). At this point, each volunteer received dental prophylaxis once again, followed by the application of aPT as previously described. The protocol for the application of aPT was similar to that used by Soukos et al.,^9^ with a home-whitening device. Following the application of aPT, patients were reviewed monthly until the BS score returned to the initial value. This period was called Restoration Period (RP). In each visit, the BS value was calculated and photographs taken for each patient as previously described. At the end of the restoration period study black plaque samples were collected according to the previously described procedure. A flow chart of the study protocol appears in Fig. 1.

### Data analysis

BS color changes were evaluated monthly and scored as follows: *score 0* (no BS), *score 1* (BS color depth post-aPT is lower than pre-aPT), *score 2* (BS color depth post-aPT is equal or greater than pre-aPT). The area covered by BS was assessed monthly and scored as follows: *score 0* (no BS); *score 1* (BS area post-aPT is smaller than pre-aPT); *score 2* (BS area post-aPT is equal to that of pre-aPT); *score 3* (BS area post-aPT is greater than that of pre-aPT). Q-RTPCR, using DNA extracted from the BS plaque samples, was carried out (Laboratorios Origen, Madrid España) to determine differences in relative proportions of *A*. *actinomycetemcomitans*, *P*. *gingivalis, and T. forsythia*, prior to and following aPT.

### Statistical analysis

The reproducibility of the measurement method was validated by a test-retest study, whose correlation was assessed by the Kappa index. The Wilcoxon test for related samples was employed to compare the CP and the RP. The McNemar test for related samples was used to evaluate the differences in time taken to reappearance of the BS pre- and post-aPT; differences in BS color and coverage area under the test conditions; and the variations in the colonization of the BS samples by the bacteria evaluated in the study. The level of significance for bivariate analysis was established at 5% (*α* = 0.05).

## Results

A total of 31 patients successfully completed the study. Five patients were lost to follow up. A further two patients were later excluded as one no longer met the study inclusion requirements and the second failed to comply with the established protocol.

### Validation of the BS measurement methodology

**Fig. 2:**
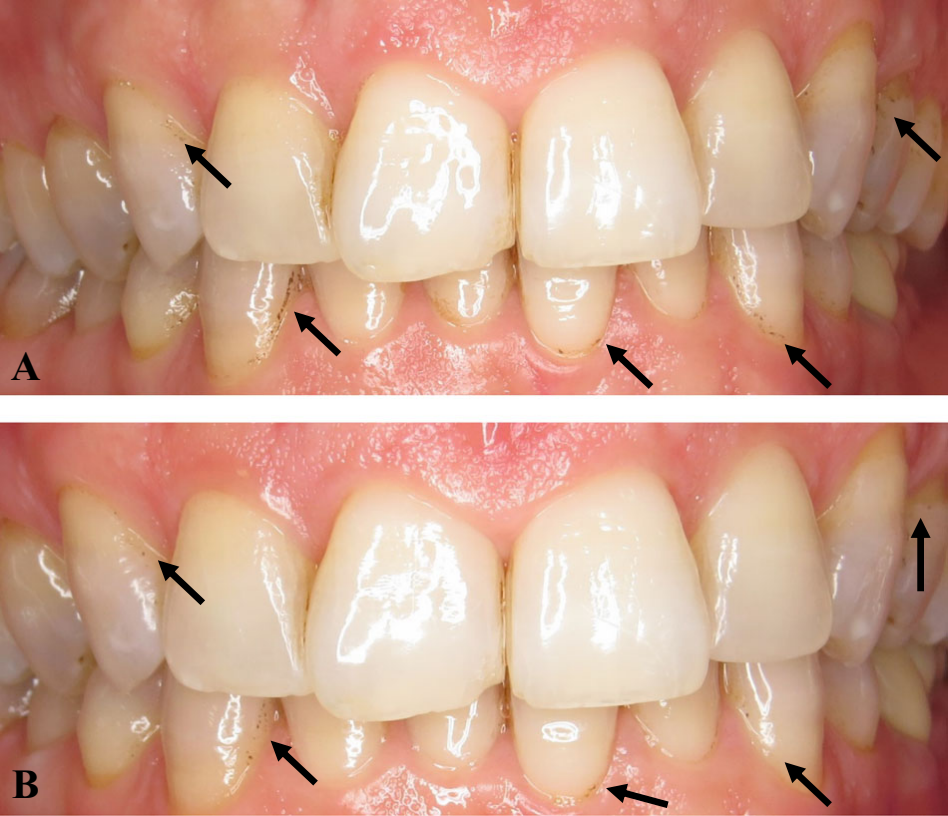
Intraoral photographs showing the distribution of black staining on a study volunteer’s teeth. Black arrows indicate the location of the stain. **a** Before prophylaxis and aPT. **b** After prophylaxis and aPT

A test-retest study was carried out according to the following protocol: Ten patient photographs from the total pool of study photographs were selected randomly by an external operator. Figure 2 shows the distribution of black stain in a study patient before and after dental prophylaxis and aPT. BS analysis was carried out on the 10 images according to the protocols described earlier. Following a 10 day period the same measurements were once again carried out on the same images. A final series of measurements were carried out after a further 15 day period. The Kappa value ranges from 0 to 1, with 0 being no agreement and 1 maximum agreement. The kappa index for intra-operator variability was calculated to be 0.855, indicating a high degree of inter-measurement reproducibility.

### Modification of the time to reappearance of BS due to aPT

The average CP was 5.3 months, whilst the average RP was 5.2 months. The Wilcoxon test of the comparison of distributions for repeated samples returned a value of 0.762 (*p* > 0.05), thus indicating that the application of aPT did not statistically significantly alter the period of time required for the re-establishment of BS to pre-treatment levels.

### Modification of the area of coverage of BS due to aPT

**Fig. 3:**
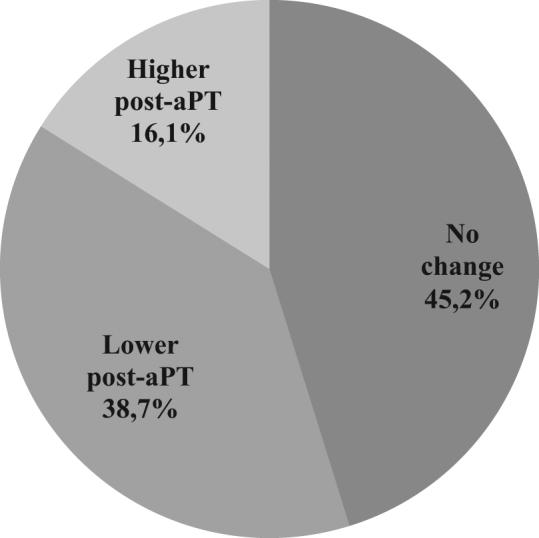
Modification of the area covered by BS in the study volunteers. Pre-aPT versus post-aPT values were compared and the differences are described as higher or lower, post-aPT therapy

The extent of BS coverage pre- and post-aPT was evaluated. Our results indicate that 45.2% of patients displayed no significant change in BS-covered area post-aPT, 38.7% experienced a reduction in BS coverage, whilst 16.1% of the patients exhibited an increase in BS surface area (Fig. 3). The result of marginality test returns a value of *p* = 0.008 (*p* < 0.05) indicating that the results were found to be statistically significant. aPT appeared to significantly reduce the coverage of BS in a minority of participants. The majority exhibited no significant difference to pre-aPT values.

### Modification of BS color due to aPT

Following prophylaxis applied during the first visit, the BS color was restored to the original level in all patients (data not shown). Following the application of aPT, data analysis reveals that BS attained a lighter-than-initial color in 26% of patients, whilst BS color returned to pre-aPT levels in 74% of patients. The McNemar test returned a P-value of 1 × 10^−4^ (*p* < 0.05), indicating that the change was found to be statistically significant. These results show that aPT causes a statistically significant initial decrease in BS color depth.

### Modification of bacterial levels in BS due to aPT

**Fig. 4:**
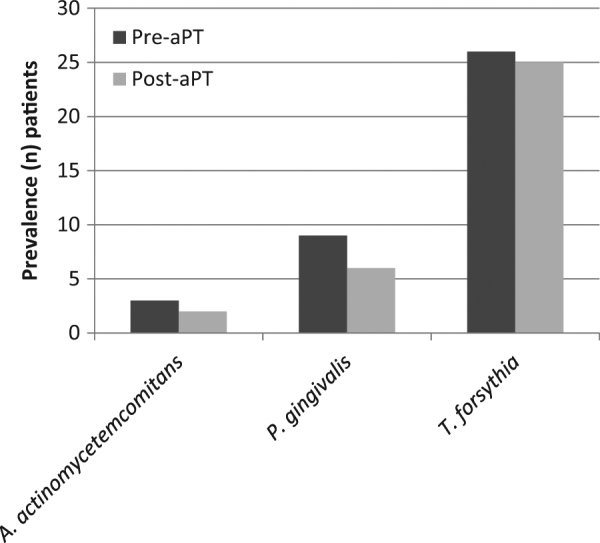
Graph showing the number of patients carrying *A. actinomycetemcomitans, P. gingivalis*, and *T. forsythia* in adult black stain pre-aPT and post-aPT

Bacterial analysis revealed that *T. forsythia* was found to be the predominant microorganism in the majority of patients with BS (*n* = 26; 83.9%), followed by *P. gingivalis* (*n* *=* 9*;* 29.03%) and *A. actinomycetemcomitans* (*n* = 3; 9.7%). Post-aPT, the presence of the three bacteria decreased *T. forsythia* (*n* = 25; 80.6%), *P. gingivalis* (*n* = 6; 19.4%), and *A. actinomycetemcomitans* (*n* = 2; 6.5%) (Fig. 4). This change was not found to be statistically significant (p > 0.05). aPT was found to non-significantly decrease the levels of bacterial species tested.

### Reporting of adverse effects following aPT

Of the 31 study patients, one reported dental hypersensitivity within fifteen days after receiving aPT. There were no reports of pain, irritation or intraoral discomfort associated with the procedure.

## Discussion

aPT is defined as the inactivation of cells, microorganisms or molecules induced by light.^9–11^ The use of aPT for the treatment of localized infections produced by bacteria is a promising therapeutic avenue for the future.^9^ This therapy has demonstrated numerous advantages in dentistry, such as rapid and painless application, selectivity for pathogenic microorganisms and the absence of side-effects in host cells.^9^

Recent studies have employed photosensitizing agents as coadjuvants to aPDT in dentistry.^17–19^ Photosensitizing agents are absorbed by microbial cells and, following exposure to light are activated, increasing the effectiveness of the aPDT through the generation of active oxygen and nitrogen free radicals. Other authors however have demonstrated effective clearance of periodontal bacteria in the absence of photosensitizing agents.^9–11,20–25^ In our study, we elected to apply aPT directly in the absence of photosensitizing agents in order to evaluate a protocol that could be simply and safely applied in a conventional dental clinic setting.

To the extent of our knowledge, this is the first formal study designed to investigate changes in black plaque over time in adult patients. Previous studies have employed laser illumination devices to deliver the light (between 415 and 495 nm) to the patients’ oral cavity.^26^ The use of laser light sources enables the investigator to control the quantity and quality of the illumination to a greater degree of precision; however this source is typically more difficult to apply simultaneously to all the teeth in the dental arch, and is not regularly found amongst the equipment in a conventional dental clinic. Our study employed a simple, commercially available device emitting blue light (470 nm), generally used for home tooth whitening. The results from our study revealed a reduction in bacterial levels in black plaque, however the magnitude of decrease was found to be greater in the study published by Soukos et al.^9^ The discrepancy may be due to differences in the power and stability of the light sources used.

The area covered by BS varies greatly between patients. Our observations coincide with previous studies, showing that individuals with good gingival health and correct oral hygiene exhibited large areas of BS (on both buccal and lingual surfaces) whereas young individuals with poor oral conditions (including periodontal disease) displayed lower areas of coverage of BS.^27–29^ During the first month after aPT 64.5% of the patients showed a reduction in pigmented area following application of aPT. Over time, BS returned to pre-aPT levels. We were not able to achieve a long-lasting eradication of BS as demonstrated in another study.^4^ Several differences between the two studies may contribute to this observation; the study published by Pessoa et al. described a *pediatric patient*, employed laser illumination, coupled with a photosensitizing agent, with the aPDT delivered in five separate occasions and supported by manual scaling. The authors indicate that they were able to achieve complete eradication of the black stain, at the cost of damage to the enamel and increased dental sensitivity in the patient, neither of which was experienced by patients in our study.

To the best of our knowledge, to date, no scientific studies have been published which investigate changes in color of BS during a therapeutic intervention. Our data show that BS regenerated, early during the restoration phase was of a lighter shade in 48.4% of the patients following aPT. Over time, the color of the BS returned to pre-aPT levels. The gradual increase in color supports the theory that the formation of BS is a biological process, dependent on microbial metabolism.

Evidence from the literature suggests differences in the microbial composition of black plaque as compared to white plaque,^5–7^ although it must be noted that these, and all published works that we could obtain, refer to studies in pediatric patients. In children studies demonstrate the presence of *A. actinomycetemcomitans* ranging from 21 to 70% in BS plaque samples.^5,6^ In our study, we show that *A. actinomycetemcomitans* was only present in 9.1% of adult BS plaque samples. In pediatric patients, *P. gingivalis* was detected in 0–34% in BS,^5,6^ consistent with our data from adult BS plaque samples (29%) of patients in our study. *Tannerella* species have recently been described for the first time in black plaque in *pediatric patients* (>1% prevalence).^7^ The striking result from our study shows the elevated prevalence of the periodontal pathogen *T. forsythia* (83.9%), suggesting a potential role for this organism in adult BS formation. Levels of all bacteria tested, decreased following aPT treatment, although the reduction was not found to be statistically significant.

Our study describes the novel application of a home whitening device for the reduction of BS in adult patients. We describe consistently positive (although minor) changes with respect to the area, color and bacterial colonization of black plaque. The application of the light source, using commercially available technology, without the use of photosensitizing agents, may increase its appeal for use in the dental clinic. We highlight the one-time application of the therapy and the low power illumination as potential areas for improvement in future studies with the aim of achieving a permanent eradication of BS.
